# Effect of Pycnogenol® on attention-deficit hyperactivity disorder (ADHD): study protocol for a randomised controlled trial

**DOI:** 10.1186/s13063-017-1879-6

**Published:** 2017-03-28

**Authors:** Annelies A. J. Verlaet, Berten Ceulemans, Helene Verhelst, Dirk Van West, Tess De Bruyne, Luc Pieters, Huub F. J. Savelkoul, Nina Hermans

**Affiliations:** 1 Department of Pharmaceutical Sciences, Laboratory of Nutrition and Functional Food Science, University of Antwerp, Universiteitsplein 1, 2610 Wilrijk, Belgium; 2 Neurology, University Hospital Antwerp, Wilrijkstraat 10, 2650 Edegem, Belgium; 3 Paediatric Neurology, University Hospital Ghent, De Pintelaan 185, 9000 Gent, Belgium; 4 Hospital Networtk Antwerp, Universitary Child and Adolescent Psychiatry, Lindendreef 1, 2020 Antwerp, Belgium; 5 Cell Biology and Immunology Group, Wageningen University, De Elst 1, 6709 PG Wageningen, The Netherlands

**Keywords:** ADHD, ADD, Behaviour, Pycnogenol®, Antioxidant, Polyphenols, Oxidative stress, Immunity, Catecholamines

## Abstract

**Background:**

Methylphenidate (MPH), the first choice medication for attention-deficit hyperactivity disorder (ADHD), is associated with serious adverse effects like arrhythmia. Evidence on the association of ADHD with immune and oxidant-antioxidant imbalances offers potential for antioxidant and/or immunomodulatory nutritional supplements as ADHD therapy. One small randomised trial in ADHD suggests, despite various limitations, therapeutic benefit from Pycnogenol®, a herbal, polyphenol-rich extract.

**Methods:**

This phase III trial is a 10-week, randomised, double-blind, placebo and active treatment controlled multicentre trial with three parallel treatment arms to compare the effect of Pycnogenol® to MPH and placebo on the behaviour of 144 paediatric ADHD and attention-deficit disorder (ADD) patients. Evaluations of behaviour (measured by the ADHD-Rating Scale (primary endpoint) and the Social-emotional Questionnaire (SEQ)), immunity (plasma cytokine and antibody levels, white blood cell counts and faecal microbial composition), oxidative stress (erythrocyte glutathione, plasma lipid-soluble vitamins and malondialdehyde and urinary 8-OHdG levels, as well as antioxidant enzyme activity and gene expression), serum zinc and neuropeptide Y level, urinary catecholamines and physical complaints (Physical Complaints Questionnaire) will be performed in week 10 and compared to baseline. Acceptability evaluations will be based on adherence, dropouts and reports of adverse events. Dietary habits will be taken into account.

**Discussion:**

This trial takes into account comorbid behavioural and physical symptoms, as well as a broad range of innovative immune and oxidative biomarkers, expected to provide fundamental knowledge on ADHD aetiology and therapy. Research on microbiota in ADHD is novel. Moreover, the active control arm is rather unseen in research on nutritional supplements, but of great importance, as patients and parents are often concerned with the side effects of MPH.

**Trial registration:**

Clinicaltrials.gov number: NCT02700685. Registered on 18 January 2016. EudraCT 2016-000215-32. Registered on 4 October 2016.

**Electronic supplementary material:**

The online version of this article (doi:10.1186/s13063-017-1879-6) contains supplementary material, which is available to authorized users.

## Background

Attention-deficit hyperactivity disorder (ADHD) is a common neurocognitive behavioural disorder with childhood onset and core symptoms of hyperactivity, impulsivity and inattention [1]. ADHD has a worldwide prevalence of 5.9–7.1% and is associated with other psychiatric disorders, such as oppositional defiant disorder (ODD), autism and anxiety [2, 3].

Methylphenidate (MPH), the first-choice medication for ADHD, is a central nervous system stimulant. It increases attentiveness and reduces hyperactivity and impulsivity by inhibition of dopamine reuptake in the striatum, without triggering its release. MPH is prescribed for chronic use to a large proportion of ADHD patients, but is linked to possible publication bias in reported efficacy [4–6]. In addition, parents are often disinclined to use MPH due to its negative publicity and its frequent side effects, including serious side effects like arrhythmia, and, subsequently, nonadherence to therapy is high [4–7]. A recent review reports adverse effects, like insomnia and decreased appetite, in about 25% of patients using MPH [8]. Other therapeutic options are therefore warranted, at least for a subgroup of patients [4, 5, 7, 8].

ADHD is a complex and multifactorial disorder, influenced by both genetics and the environment. Its exact pathophysiology remains, however, unclear. Dopaminergic dysfunction is involved, but also associations with immune and oxidant-antioxidant imbalances exist [9, 10]. Various studies demonstrated, for example, increased levels of plasma malondialdehyde (MDA) and exhalant ethane (oxidative stress markers) and decreased activity of antioxidant enzymes such as glutathione peroxidase (GPX) and catalase (CAT) [11–14]. ADHD has also been hypothesised to be a hypersensitivity disorder, with a disrupted immune regulation contributing to its aetiology [10]; i.e. ADHD has comorbidity with both Th1- and Th2-mediated disorders and several related genes have immune functions [9, 10, 15–18]. Ceylan et al. observed increased levels of adenosine deaminase, a marker of cellular immunity, and of the oxidative enzymes xanthine oxidase (XO) and nitric oxide synthase, and decreased levels of the antioxidant enzymes glutathione-*S*-transferase and paraoxonase-1. These results indicate the involvement of oxidative changes and cellular immunity in ADHD [9].

Still, specific immune biomarkers other than antibodies have not been systematically studied in ADHD despite growing evidence on associations in autism [19, 20]. In addition, immune and oxidative effects of both standard therapy and nutritional supplementation in ADHD are neglected topics in research. Yet, immune and oxidative imbalances linked with ADHD offer potential for appropriate supplementation in ADHD therapy [21].

Due to its antioxidant and immunomodulatory properties, a commercially available standardised extract from French maritime pine (*Pinus pinaster*) bark with a high content of polyphenolic compounds (including phenolic acids and procyanidins), Pycnogenol®, was selected for this study [22–24]. One small randomised trial and few observational studies suggest its therapeutic benefit in ADHD. Still, this trial had some limitations (e.g. short supplementation period) and the mechanisms of action involved remain unclear [22, 25–29]. The efficacy, mechanism(s) of action and value of Pycnogenol® in ADHD as compared to MPH treatment thus remain to be confirmed.

## Methods

### Objective

To evaluate the effect of Pycnogenol® on ADHD and ADD behaviour and comorbid physical and psychiatric symptoms, as well as on immunity, oxidative damage, antioxidant status and neurochemical parameters, as compared to placebo and MPH treatment.

### Hypotheses

1. In ADHD therapy, Pycnogenol® is more effective than placebo and not less effective than MPH
2. As compared to placebo and MPH, Pycnogenol® increases antioxidant levels, reduces oxidative damage, improves immune and neurochemical status and reduces comorbid physical and psychiatric complaints
3. The tolerability of Pycnogenol® is higher than that of MPH

### Design

This is a phase III, randomised, double-blind, placebo and active product controlled, multicentre clinical trial with three parallel treatment arms to compare effects on ADHD and ADD behaviour between Pycnogenol®, MPH (Medikinet® Retard) and placebo, using the ADHD-Rating Scale (ADHD-RS) as a primary outcome measure. Secondary outcome measures are comorbid physical and psychiatric complaints (including side effects), oxidative stress, immunity, neurochemical parameters and tolerance of the intervention. Following screening and baseline assessments, 144 patients (aged 6–12 years) will receive one of the three treatments for 10 weeks (see Table 2). Evaluations will be performed in weeks 5 and 10, as compared to baseline. Dietary habits will be taken into account.

Two visits with similar evaluations and sample collections will be conducted: at baseline and after 10 weeks. To analyse biomarkers of interest, 16 ml of venous blood will be collected at the start and the end of intervention, as well as urine. Faecal samples will be collected from participant subgroups (*n* = 60). Next to baseline and final evaluations, an extra evaluation of behaviour and physical symptoms will be conducted in week 5 by means of questionnaires. Two reminders will be sent in case questionnaires are not received within 1 week after the required date. After every blood and urine collection and when questionnaires are completed, participants will receive two movie tickets.

### Inclusion and randomisation

Recruitment starts in March 2017. The trial population will consist of ADHD and ADD patients recruited at the University Hospitals of Antwerp (UZA) and Ghent (UZ Ghent) and the Hospital Network Antwerp (ZNA). With an expected inclusion rate of 30–50 patients per year (10–20 participants in UZA, 15–20 in UZ Ghent and 5–10 in ZNA), about 3 years will be required for subject recruitment. Though, as compared to inclusion rate, a ten-fold higher diagnosis rate of ADHD and ADD is expected in these centres, inclusion and exclusion criteria of the proposed trial (e.g. regarding autism or the recent intake of supplements or medication) are expected to exclude at least half of all newly diagnosed patients, while a consent rate of 30% is expected, taking into account potential reluctance regarding the use of medication or supplements, as well as ‘risk’ for placebo treatment [30, 31]. In addition, patients from random primary schools in Flanders will be invited for this trial by letters and diagnosed in one of the trial centres before inclusion. In case of slow recruitment, also ‘ZitStil’ (information centre on ADHD/ADD), revalidation centres, independent child psychiatrists/paediatricians and other hospitals may be involved. Patients meeting eligibility criteria (Table 1) will be informed in detail and written consent of the legal representative to participate in the trial will be obtained prior to inclusion.

**Table 1:** Inclusion and exclusion criteria for patient selection

Inclusion criteria	Exclusion criteria
1. Age 6–12 years (both inclusive)	1. Diagnosis of autism spectrum disorder
2. ADHD diagnosis a. based upon the diagnostic interview by the investigating physician b. based upon the ADHD-RS	2. Pervasive developmental disorder, personality disorder, IQ <70, conduct disorder (CD), tics, schizophrenia, dyskinesia, personal or family history of psychotic disorder, bipolar illness, depression, or suicide attempt
3. Responsible caregiver to provide information about the patient’s functional status	3. Chronic medical disorder or acute inflammatory disease. Glaucoma, heart disease, high blood pressure, or peripheral vascular disease
4. Patient and responsible caregiver have a sufficient level of knowledge of Dutch	4. Use of MAO inhibitor 14 days before inclusion. Use of clonidine, guanethidine, seizure medicine, antidepressants, blood thinners, blood pressure, or diet medication 3 months before inclusion
5. Written informed consent by the patient’s legally accepted representative	5. Use of vitamin/mineral/herbal/omega-3 supplements or any medication for longer than 1 week, 3 months before inclusion
	6. Other contraindications for MPH or Pycnogenol®, as defined in the Summary of Product Characteristics and Investigator’s Brochure, respectively

*ADHD* attention-deficit hyperactivity disorder, *ADHD-RS*, ADHD-Rating Scale, *MAO* monoamine oxidase, *MPH* methylphenidate

**Fig. 1:**
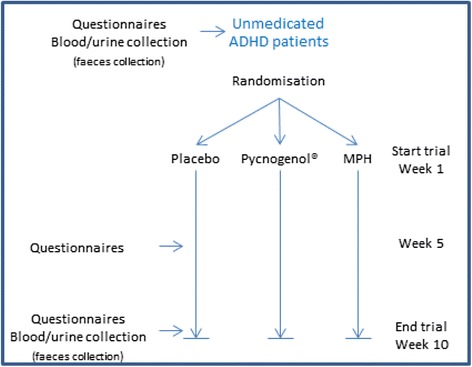
Design of the trial

Participants will be randomised, stratified by trial centre, to one of the three treatment arms (placebo, Pycnogenol®, or Medikinet® Retard) by randomization.com randomisation software (original generator, different starting number across trial sites, and taking into account body weights below and above 30 kg; Fig. 1). The number of patients per trial site is not limited. The involved physicians and hospital pharmacies will assure confidentiality by retaining the randomisation code at all times in a sealed envelope, only to be used in case of emergency or serious adverse events.

### Treatment

Patients will receive all capsules required for the complete study at inclusion, at a dose based on their body weight (one or two orally administered capsules at breakfast):

- MPH (Medikinet® Retard, methylphenidate hydrochloride modified release): patients with a body weight below 30 kg will receive 20 mg/day, those with a body weight of 30 kg or over, 30 mg/day. Treatment during the first week always contains 10 mg, increasing by 10 mg per week to limit side effects
- Pycnogenol®: patients with a body weight below 30 kg will receive 20 mg/day, those with a body weight of 30 kg or over, 40 mg/day, aiming at a daily dose of 1 mg/kg and taking into account formulation issues [22]. Treatment during the first 2 weeks always contains 20 mg
- Placebo: contains excipients only

In case of adverse events, the investigator, principal caregiver and participant can decide to discontinue the trial medication/supplement. However, no dose adjustment will be performed. Using a standardised questionnaire, adverse events will be documented at weeks 5 and 10, also taking into account the patient’s medical records. Additionally, spontaneously reported adverse events will be recorded. In case of a serious adverse event, the trial code will be broken and treatment discontinued. In case 10% of participants experience a potentially related serious adverse event, the trial will be discontinued.

Pycnogenol® and placebo will be produced in capsules identical to Medikinet® Retard (Medice GmbH). All treatments will be provided in identical jars, labelled with the subject’s trial number and week of intake. Compliance will be determined based on accountability of investigational products and self-reported adherence.

### Primary outcome

As the primary objective is to assess the efficacy of Pycnogenol® for improving ADHD and ADD behaviour as rated by teachers compared to placebo and Medikinet® Retard, the primary outcome is the summed ADHD score of the ADHD-RS as rated by teachers (Table 2). Teachers will fill out this questionnaire before the start of the intervention and after 5 and 10 weeks.

**Table 2:** Investigations and data acquisition during the trial

	Screening	Baseline		
Evaluations/interventions	Week 0	Week 0	Week 5	Week 10
Inclusion and exclusion criteria	X	X		
Current use of medication/supplements	X	X	X	X
Informed consents		X		
Randomisation		X		
Treatment				
Treatment distribution		X		
Medication count		X		X
ADHD-RS		X	X	X
SEQ		X		X
PCQ		X	X	X
FFQ		X		X
Blood and urine collection		X		X
Faeces collection		X		X
GSH analysis		X		X
Lipid-soluble antioxidants analysis		X		X
Antioxidant enzyme activity		X		X
Genetics analysis		X		X
MDA analysis		X		X
8-OHdG analysis		X		X
Cytokine analysis		X		X
Antibody analysis		X		X
PBMC count and reactivity analysis		X		X
Microbial composition analysis		X		X
Catecholamine analysis		X		X
NPY analysis		X		X
Zinc analysis		X		X

*8-OHdG* 8-hydroxy-2-deoxyguanosine, *ADHD-RS* ADHD-Rating Scale, *FFQ* Food Frequency Questionnaire, *GSH* erythrocyte glutathione*, MDA* plasma malondialdehyde, *NPY* serum neuropeptide Y, *PBMC* peripheral blood mononuclear cell, *PCQ* Physical Complaints Questionnaire, *SEQ* Social-Emotional Questionnaire

### Secondary outcomes

ADHD/ADD behaviour Secondary outcomes related to ADHD/ADD behaviour are:

- Summed ADHD score of the ADHD-RS, rated by parents
- Summed ADHD score of the Social-emotional Questionnaire (SEQ), rated by parents and teachers
- Scores on ADHD subscales of the ADHD-RS and SEQ, rated by parents and teachers (hyperactivity, impulsivity and inattention)
- Percentage of responders rated by parents and teachers, defined as participants with a reduction of at least 20% of their baseline summed ADHD-RS score [32]

Other objectives are to evaluate the effect of Pycnogenol® compared to placebo and MPH on comorbid psychiatric and physical complaints, antioxidant levels, oxidative damage, immunity and neurotransmitters. Other secondary outcomes are, therefore:

#### Psychiatric complaints

- Social behaviour problems subscale of the SEQ, rated by parents and teachers, to evaluate to what extent symptoms of ODD and CD are displayed [33]
- Anxiety subscale of the SEQ, rated by parents and teachers, to evaluate symptoms of general anxiety, social anxiety and anxiety-depression [33]

#### Physical complaints

- Physical and sleep complaints, including various potential side effects, measured by the Physical Complaints Questionnaire (PCQ) [34]

#### Antioxidant levels

- Erythrocyte glutathione (GSH) level, the most important intracellular antioxidant, analysed by HPLC with electrochemical detection [35]
- Lipid-soluble antioxidants: plasma vitamin E (α- and γ-tocopherol), vitamin A (β-carotene, retinol, retinyl palmitate) and co-enzyme Q10, analysed by HPLC with coulometric detection [36–39]
- Antioxidant enzyme activity (CAT, SOD and GPX) and total antioxidant status, analysed by Enzyme-linked ImmunoSorbent Assay (ELISA) [11, 40]
- Gene expression, quantified by RT-qPCR, focusing on networks counteracting oxidative stress (GPX, CAT, superoxide dismutase (SOD), XO) and stress-related proteins (clusterin, apolipoprotein J) [41, 42]
- Serum zinc level, analysed by atomic absorption spectroscopy (AAS) [43]

#### Oxidative damage

- Urinary 8-hydroxy-2'-deoxyguanosine (8-OHdG) level, marker of oxidative deoxyribonucleic acid (DNA) damage, corrected for urinary creatinine concentration, analysed by ELISA [44]
- Plasma malondialdehyde (MDA) level, marker of lipid peroxidation, analysed by HPLC with fluorescence detection [45]

#### Immunity

- Plasma cytokines for monocytes (interleukin (IL)-1β, IL-6, IL-8, IL-10, IL-12, tumour necrosis factor (TNF)-α) and T-cells (IL-4, IL-5, IL-6, IL-10, interferon (IFN)-γ) as well as antibody levels (IgA_1–2_, IgG_1–4_, IgE) by flow cytometry and ELISA, as markers of immune activation state and skewing [21, 46, 47]
- Identification of peripheral blood mononuclear cells (PBMCs) like neutrophils, CD4, CD8 and B-cells and measurement of their functional responses (e.g. cytokine release) after stimulation, as a marker of immune activation state, skewing and responsivity [21, 46, 47]
- Intestinal microbial composition, assessed using extreme throughput multiplexed sequencing of 16S ribosomal ribonucleic acid (RNA) gene pools polymerase chain reaction (PCR)-amplified from intestinal content samples [48, 49]

#### Neurochemistry

- Urinary catecholamines (dopamine, noradrenaline and adrenaline) and their metabolites, determined by HPLC with coulometric detection [50]
- Serum neuropeptide Y (NPY), analysed by ELISA [51]

The final objective is to investigate the acceptability of Pycnogenol® compared to Medikinet® Retard and placebo, based on the prevalence of side effects, treatment adherence (defined as more than 90% ingestion as scheduled) and proportion of dropouts.

Dietary habits of participants, such as consumption of vegetables, chocolate, fruit, etc., will be assessed by a Food Frequency Questionnaire (FFQ) at the start and end of intervention [52, 53], to assess potential dietary adaptations during the study as well as baseline differences between treatment groups. The highest educational achievement of both parents will be determined as a proxy for socioeconomic status.

### Statistics

For the estimation of the required sample size, the following assumptions were made:

- Patients improve by 0.75 standard deviation (SD) on the ADHD-RS summed ADHD score as rated by teachers if using Pycnogenol® for 10 weeks [22, 54]*,* which corresponds to a 20% improvement with active treatment as compared to placebo
- Power of 80%, dropout of 20%
- Two-sided testing, at a significance level of 0.05 with Bonferroni post-hoc testing correction

Based on these considerations, 48 patients per group will be necessary (*n* = 144 in total).

Data will be checked for outliers. Missing data will not be accounted for. The three groups will be compared with regard to baseline characteristics. A two-way analysis of variance (ANOVA) will be performed to investigate a potential interaction between treatment and weight. Change in ADHD-RS score as rated by teachers (primary outcome measure) will be compared between the three groups by means of a one-way ANOVA (categories: group, time; *α* = 0.05) with post-hoc testing. Changes regarding secondary target variables will also be compared between the three groups, by one-way ANOVA with post-hoc analysis with multiple testing correction, Kruskal-Wallis, or Fisher’s exact test. Separate analyses for subgroups (e.g. based on gender, severity of ADHD, dietary habits, etc.) will be performed. Noninferiority of Pycnogenol® as compared to Medikinet® Retard will be demonstrated when the difference in effect on ADHD-RS score is no more than 5 points [55]. This wide margin might be justified due to frequent side effects of MPH. Noninferiority will only be accepted if supported by both intention-to-treat and per-protocol analyses [56, 57].

### Ethics and registration

Ethical approval has been obtained in UZA (EC 15/35/365), ZNA (EC approval 4656) and UZ Ghent (2016/0969). The trial has been registered at ClinicalTrials.gov (NCT02700685) and EudraCT (2016-000215-32).

#### Trial management and research team

The University of Antwerp (Laboratory of Nutrition and Functional Food Science) is the sponsor of this trial, with NH being the coordinating investigator. As principle investigators, BC, DVW and HV will be primarily responsible for patient inclusion. NH and AV are responsible for the analysis of oxidative stress and neurological biomarkers and questionnaire results, as well as data management. HS is responsible for the analysis of immune biomarkers and genetics. No Data Monitoring Committee will be set up. BC, HV, DVW, NH and AV will discuss potential issues regarding, e.g. subject recruitment.

For more information, see both the Standard Protocol Items: Recommendations for Interventional Trials (SPIRIT) Checklist and figure (Additional file 1 and Table 2), with more detailed information on the execution of the trial, and scientific, ethical and administrative elements.

## Discussion

This randomised controlled trial addresses the therapeutic potential of a herbal extract in ADHD by investigating its efficacy, mechanism of action and value as compared to standard treatment and placebo. Results can be partly compared to a previously conducted study [22, 28, 29]. A double-blind design was chosen to avoid bias, due to the subjectivity of questionnaire responses. Behavioural assessment by teachers is preferred as primary objective due to the higher sensitivity of teachers’ ratings [58, 59]. A 10-week treatment is considered long enough to see clear effects of both Pycnogenol® and Medikinet® Retard, though still minimising the patient burden and thus maximising compliance [22]. The parallel design was, therefore, also chosen to reduce patient burden.

Pycnogenol® is a patented, proprietary powder extract made exclusively from French maritime pine bark by Horphag Research (Geneva, Switzerland). The extract is standardised to contain 70 ± 5% procyanidins. Pharmacological studies employing in vitro, animal and/or human models have found potent antioxidant activity, anti-inflammatory actions, improvement of endothelial function, etc. [60]. Pycnogenol® was selected for the present study based on previous research suggesting its therapeutic benefits in ADHD, though this trial had several limitations [22, 27–29]. Further research is needed to investigate its efficacy, mechanism of action and value, especially compared to MPH treatment. For example, dietary polyphenols and their metabolites exert prebiotic-like effects, stimulating the growth of intestinal microbiota, which play a fundamental role in immunity [48, 61, 62]. Also the Pycnogenol® dosage is based upon this previous clinical trial, using 1 mg/kg body weight [22]. In the present trial, due to practical reasons, 0.67–1.33 mg/kg body weight will be applied.

Despite being the first choice medication for ADHD, MPH is associated with various adverse effects (including serious adverse events), some of them frequently occurring, including irritability, insomnia, loss of appetite and headache [8]. Based on data from 70 human clinical studies on 5723 healthy subjects and patients, the overall frequency of adverse side effects due to Pycnogenol® is very low (1.8%) and unrelated to dose or duration of use. The majority of adverse effects observed are mild. Gastrointestinal discomfort, the most frequently occurring adverse effect, may be avoided by taking Pycnogenol® with or after meals. In children with ADHD, 2 of 41 Pycnogenol®-supplemented participants experienced side effects (rise of slowness and moderate gastric discomfort). Pycnogenol® did not cause any significant changes in blood pressure or heart rate in four clinical studies (total *n* = 185). There have been no reports of serious adverse effects since its introduction into the European market around 1970 [49]. Safety trials have demonstrated the absence of mutagenic and teratogenic effects, no perinatal toxicity and no negative effects on fertility [63]. Therefore, the use of Pycnogenol® in children is considered to be safe.

The ADHD-RS is validated and internationally accepted, and consists of nine inattention and nine impulsivity and hyperactivity items based on the *Diagnostic and Statistical Manual of Mental Disorders* (DSM), each marked out on a four-point rating scale [23]. The ADHD-RS allows comparison of results to those of previously performed trials [22, 54].

In addition to the ADHD-RS, the SEQ is used in this trial. Though this increases the number of questions on behaviour significantly (72 questions), the SEQ is a behaviour evaluation list to assess core symptoms of social-emotional problems, including frequently occurring psychiatric comorbidities of ADHD. Besides ADHD, three other clusters of social-emotional problems are incorporated in the SEQ (social behaviour problems, anxiety and autism), with items covering the core symptoms of these clusters according to DSM. The SEQ can be used for screening, diagnosis and treatment evaluation. Items are rated on a five-point scale [33].

The approved PCQ consists of 36 questions, of which 18 items are relevant with respect to specific physical and sleep complaints, including eight domains: (1) pain (e.g. headache), (2) unusual thirst or perspiration, (3) eczema, (4) asthma or rhinitis, (5) skin problems (e.g. blotches in the face), (6) tiredness, (7) gastrointestinal problems and (8) sleep problems. Items, including various potential adverse effects, are rated on a five-point scale at baseline and after 5 and 10 weeks [34]. In addition, parents will be asked whether the participant experienced any illness during the trial, what illness, whether any medication was taken, and the type, dose and duration of medication intake.

The FFQ consists of 50 questions on different food groups to be rated on a nine-point scale by parents at the start and end of the intervention, to assess baseline dietary habits and potential adaptations during the study, as well as to relate potential differential effects of Pycnogenol® to dietary polyphenol intake [52, 53]. Insight in global dietary habits (e.g. whether or not the participant consumes fresh fruit on a daily basis) is, therefore, aimed for.

Patients and especially their parents are often worried about side effects of MPH, the standard medication for ADHD. It is, therefore, important to take into account side effects of Pycnogenol® and effects on comorbid complaints. In addition, the behavioural effects of Pycnogenol® compared to placebo, but also compared to MPH, will be investigated. This active control is rather unseen in research on nutritional supplements, but of great importance. In one previous trial, the effect of Pycnogenol® was compared to MPH and placebo. However, neither MPH nor Pycnogenol® outperformed placebo, possibly due to the short treatment period of 3 weeks [27].

Most research on nutritional supplements or medication in ADHD predominantly assesses effects on ADHD behaviour. This trial, however, takes into account comorbid behavioural and physical symptoms, such as ODD, anxiety and side effects, as well as a broad range of innovative immune, oxidative and neurochemical biomarkers. The analysis of gene expression and biomarkers can indicate genetic effects and biological processes involved in the mechanism of action of Pycnogenol® and possibly affecting ADHD symptom expression. Research on microbiota in ADHD in itself is novel, too. Results of this project will, therefore, increase insight in ADHD aetiology and (dietary) treatment options, which is highly desired by medical staff, parents and patients.

### Trial status

Not yet recruiting as of February 2016.

## Additional file

Additional file 1: SPIRIT 2013 Checklist: recommended items to address in a clinical trial protocol and related documents*. (DOCX 100 kb)

## Abbreviations

8-OHdG: 8-hydroxy-2-deoxyguanosine
AAS: Atomic absorption spectroscopy
ADD: Attention-deficit disorder
ADHD: Attention-deficit hyperactivity disorder
ADHD-RS: ADHD-Rating Scale
ANOVA: Analysis of variance
CAT: Catalase
DSM: *Diagnostic and Statistical Manual of Mental Disorders*
ELISA: Enzyme-linked ImmunoSorbent Assay
FFQ: Food Frequency Questionnaire
GPX: Glutathione peroxidase
GSH: Reduced glutathione
IFN: Interferon
IL: Interleukin
MAO: Monoamine oxidase
MDA: Malondialdehyde
MPH: Methylphenidate
NPY: Neuropeptide Y
PCQ: Physical Complaints Questionnaire
RT-qPCR: Real-time quantitative polymerase chain reaction
SEQ: Social-emotional Questionnaire
SOD: Superoxide dismutase
TNF: Tumour necrosis factor
UZ Ghent: University Hospital Ghent
UZA: University Hospital Antwerp
XO: Xanthine oxidase
ZNA: Hospital Network Antwerp

## References

[1] Pelham WE, Foster EM, Robb JA (2007). The economic impact of attention-deficit/hyperactivity disorder in children and adolescents. J Pediatr Psychol.

[2] Polanczyk GV (2014). ADHD prevalence estimates across three decades: an updated systematic review and meta-regression analysis. Int J Epidemiol.

[3] Biederman J, Faraone SV (2005). Attention-deficit hyperactivity disorder. Lancet.

[4] Antshel KM (2011). Advances in understanding and treating ADHD. BMC Med.

[5] Claes S (2010). Het toenemend gebruik van psychofarmaca. Visietekst werkgroep Metaforum Leuven.

[6] Schachter HM (2011). How efficacious and safe is short-acting methylphenidate for the treatment of attention-deficit disorder in children and adolescents?. A meta-analysis CMAJ.

[7] Steer CR, Suppl I (2005). Managing attention deficit/hyperactivity disorder: unmet needs and future directions. Arch Dis Child.

[8] Storebø OJ (2015). Methylphenidate for attention-deficit/hyperactivity disorder in children and adolescents: Cochrane systematic review with meta-analyses and trial sequential analyses of randomised clinical trials. BMJ.

[9] Ceylan M (2012). Changes in oxidative stress and cellular immunity serum markers in attention-deficit/hyperactivity disorder. Psychiatry Clin Neurosci.

[10] Pelsser LMJ, Buitelaar JK, Savelkoul HFJ (2009). ADHD as a (non) allergic hypersensitivity disorder: a hypothesis. Pediatr Allergy Immunol.

[11] Ceylan M (2010). Oxidative imbalance in child and adolescent patients with attention-deficit/hyperactivity disorder. Prog Neuropsychopharmacol Biol Psychiatry.

[12] El Adham EK, Hassan AI, El Aziz El-Mahdy AA. Nutritional and metabolic disturbances in attention deficit hyperactivity disease. Res J Med Med Sci. 2011;6(1):10–6.

[13] Ross BM (2003). Increased levels of ethane, a non-invasive marker of n-3 fatty acid oxidation, in breath of children with attention deficit hyperactivity disorder. Nutr Neurosci.

[14] Kawatani M, Tsukahara H, Mayumi M (2011). Evaluation of oxidative stress status in children with pervasive developmental disorder and attention deficit hyperactivity disorder using urinary-specific biomarkers. Redox Rep.

[15] Güngör S (2013). The frequency of celiac disease in attention-deficit hyperactivity disorder. J Pediatr Gastroenterol Nutr.

[16] Minter K (2001). Early childhood otitis media in relation to children’s attention-related behavior in the first six years of life. Pediatrics.

[17] Schmitt J, Buske-Kirschbaum A, Roessner V (2010). Is atopic disease a risk factor for attention-deficit/hyperactivity disorder? A systematic review. Allergy.

[18] Tsai SJ (2006). Signal transducer and activator of transcription 6 (STAT6) and attention-deficit hyperactivity disorder: a speculative hypothesis. Med Hypoth.

[19] Furlano R (2001). Colonic CD8 and gamma delta T-cell infiltration with epithelial damage in children with autism. J Pediatr.

[20] Noriega DB, Savelkoul HFJ (2014). Immune dysregulation in autism spectrum disorder. Eur J Ped.

[21] Verlaet AAJ (2014). Nutrition, immunological mechanisms and dietary immunomodulation in ADHD. Eur Child Adolesc Psychiatry.

[22] Trebatická J (2006). Treatment of ADHD with French maritime pine bark extract. Pycnogenol Eur Child Adolesc Psychiatry.

[23] D’Andrea G (2010). Pycnogenol: a blend of procyanidins with multifaceted therapeutic applications?. Fitoterapia.

[24] Wilson D (2010). A randomized, double-blind, placebo-controlled exploratory study to evaluate the potential of Pycnogenol for improving allergic rhinitis symptoms. Phytother Res.

[25] Chovanová Z (2006). Effect of polyphenolic extract, Pycnogenol, on the level of 8-oxoguanine in children suffering from attention deficit/hyperactivity disorder. Free Radical Res.

[26] Dvořáková M (2006). The effect of polyphenolic extract from pine bark, Pycnogenol, on the level of glutathione in children suffering from attention deficit hyperactivity disorder (ADHD). Redox Rep.

[27] Tenenbaum S (2002). An experimental comparison of Pycnogenol and methylphenidate in adults with attention-deficit/hyperactivity disorder (ADHD). J Atten Disord.

[28] Greenblatt J (1999). Nutritional supplements in ADHD. J Am Acad Child Adolesc Psychiatry.

[29] Heimann SW (1999). Pycnogenol for ADHD?. J Am Acad Child Adolesc Psychiatry.

[30] Jennings CG (2015). Does offering an incentive payment improve recruitment to clinical trials and increase the proportion of socially deprived and elderly participants?. Trials.

[31] Kerkhoff LA (2016). Trends in consent for clinical trials in cardiovascular disease. J Am Heart Assoc.

[32] Buitelaar JK, Montgomery SA, van Zwieten-Boot BJ (2003). Attention deficit hyperactivity disorder: guidelines for investigating efficacy of pharmacological intervention. Eur Neuropsychopharmacol.

[33] TestWeb. Sociaal-Emotionele Vragenlijst (SEV). 2013. Available from: http://testweb.bsl.nl/tests/sev/. Accessed date: 15 Dec 2016.

[34] Pelsser LMJ, Buitelaar JK (2002). Favourable effect of a standard elimination diet on the behaviour of young children with attention deficit-hyperactivity disorder (ADHD), a pilot study. Ned Tijdschr Geneesk.

[35] Pastore A (2003). Analysis of glutathione: implication in redox and detoxification. Clin Chim Acta.

[36] Conaway HH, Henning P, Lerner UH (2013). Vitamin a metabolism, action, and role in skeletal homeostasis. Endocr Rev.

[37] Naguib Y (2003). Antioxidant activities of natural vitamin E formulations. J Nutr Sci Vitaminol.

[38] Littarru GP, Tiano L. Bioenergetic and antioxidant properties of coenzyme Q10: recent developments. Mol Biotechnol. 2007;37:31–7. 10.1007/s12033-007-0052-y 17914161

[39] Molyneux SL (2008). Coenzyme Q10: is there a clinical role and a case for measurement?. Clin Biochem Rev.

[40] Sezen H (2016). Increased oxidative stress in children with attention deficit hyperactivity disorder. Redox Rep.

[41] Franceschi C (2005). Genes involved in immune response/inflammation, IGF1/insulin pathway and response to oxidative stress play a major role in the genetics of human longevity: the lesson of centenarians. Mech Ageing Dev.

[42] Rahman I, Yang SR, Biswas SK (2006). Current concepts of redox signaling in the lungs. Antioxid Redox Signal.

[43] Whitehouse RC (1982). Zinc in plasma, neutrophils, lymphocytes, and erythrocytes as determined by flameless atomic absorption spectrophotometry. Clin Chem.

[44] Aruoma OI (1998). Free radicals, oxidative stress, and antioxidants in human health and disease. JAOCS.

[45] Dalle-Donne I (2006). Biomarkers of oxidative damage in human disease. Clin Chem.

[46] Furukawa T, Meydani SN, Blumberg JB (1987). Reversal of age-associated decline in immune responsiveness by dietary glutathione supplementation in mice. Mech Ageing Dev.

[47] Grimm T, et al. Inhibition of NF-κB activation and MMP-9 secretion by plasma of human volunteers after ingestion of maritime pine bark extract (Pycnogenol). J Inflam. 2006;3(1):1–6. 10.1186/1476-9255-3-1 PMC1413525 16441890

[48] Booijink CC (2010). High temporal and inter-individual variation detected in the human ileal microbiota. Environ Microbiol.

[49] González TJB, et al. Study of the aminoglycoside subsistence phenotype of bacteria residing in the gut of humans and zoo animals. Front Microbiol. 2015;6:1–7. 10.3389/fmicb.2015.01550 PMC4707250 26793182

[50] Dvořáková M (2007). Urinary catecholamines in children with attention deficit hyperactivity disorder (ADHD): modulation by a polyphenolic extract from pine bark (Pycnogenol). Nutr Neurosci.

[51] Ozcan O, et al. Plasma leptin, adiponectin, neuropeptide Y levels in drug naive children with ADHD. J Atten Disord. 2015 [Epub ahead of print]. 10.1177/1087054715587095 26078399

[52] de Vriese S (2005). The Belgian food consumption survey: aims, design and methods. Archives Public Health.

[53] De Keyzer W (2012). Relative validity of a short qualitative food frequency questionnaire for use in food consumption surveys. Eur J Public Health.

[54] Pelsser LMJ (2011). Effects of a restricted elimination diet on the behaviour of children with attention-deficit hyperactivity disorder (INCA study): a randomised controlled trial. Lancet.

[55] Berek M, et al. Improved functionality, health related quality of life and decreased burden of disease in patients with ADHD treated with OROS® MPH: is treatment response different between children and adolescents? Child Adolesc Psych Ment Health. 2011;5(26):1–13. 10.1186/1753-2000-5-26 PMC3162502 21791096

[56] Christensen E (2007). Methodology of superiority vs. equivalence trials and non-inferiority trials. J Hepatol.

[57] Piaggio G (2006). Reporting of noninferiority and equivalence randomized trials: an extension of the CONSORT statement. JAMA.

[58] Power TJ (1998). The predictive validity of parent and teacher reports of ADHD symptoms. J Psychopathol Behav Assess.

[59] Tripp G, Schaughency EA, Clarke B (2006). Parent and teacher rating scales in the evaluation of attention-deficit hyperactivity disorder: contribution to diagnosis and differential diagnosis in clinically referred children. J Dev Behav Pediatr.

[60] Rohdewald PJ. Pycnogenol, French maritime pine bark extract. In: Coates PM, Blackman MR, Cragg GM, Levine M, Moss J, White JD, editors. Encyclopedia of Dietary Supplements. New York, Marcel Dekker, Inc.; 2005;545–53.

[61] Belkaid Y, Hand T (2014). Role of the microbiota in immunity and inflammation. Cell.

[62] Cardona F (2013). Benefits of polyphenols on gut microbiota and implications in human health. J Nutr Biochem.

[63] Rohdewald P (2002). A review of the French maritime pine bark extract (Pycnogenol), a herbal medication with a diverse clinical pharmacology. Int J Clin Pharmacol Ther.

