# Supplementary material for: Effect of Pycnogenol® on attention-deficit hyperactivity disorder (ADHD): study protocol for a randomised controlled trial
Source: Trials. 2017 Mar 28;18:145. doi: 10.1186/s13063-017-1879-6 (PMC5370458; doi:10.1186/s13063-017-1879-6)
Supplement: Additional file 1: — SPIRIT 2013 Checklist: recommended items to address in a clinical trial protocol and related documents*. (DOCX 100 kb) [file 13063_2017_1879_MOESM1_ESM.docx]

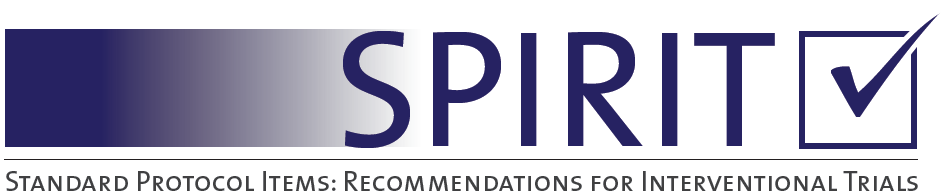


SPIRIT 2013 Checklist: Recommended items to address in a clinical trial protocol and related documents*

| Section/item | ItemNo | Description |
| --- | --- | --- |
| **Administrative information** | | |
| Title | 1 | Effect of Pycnogenol® on attention-deficit hyperactivity disorder (ADHD): A randomised, double blind, placebo and active product controlled multicentre trial. |
| Trial registration | 2a | NCT02700685  EudraCT 2016-000215-32 |
|  | 2b | Primary Registry and Trial Identifying Number: NCT02700685  Date of Registration in Primary Registry: 4 March 2016  Secondary Identifying Numbers: EudraCT 2016-000215-32, Sponsor trial number Pycno 2015-14, UZA EC 15/35/365, UZ Ghent EC 2016/0969, ZNA EC 4656.  Source(s) of Monetary or Material Support: Fund Scientific Research Flanders (FWO).  Primary Sponsor: University of Antwerp  Contact for Public Queries: Annelies Verlaet, Universiteitsplein 1 (A104), 2610 Wilrijk; annelies.verlaet@uantwerpen.be; +32 3 265 27 06  Contacts for Scientific Queries:  Berten Ceulemans, MD, Neurology, Wilrijkstraat 10, 2650 Edegem, berten.ceulemans@uza.be, +32 3 821 34 23; Annelies Verlaet, Laboratory of Nutrition and Functional Food Science, Universiteitsplein 1 (A104), 2610 Wilrijk, annelies.verlaet@uantwerpen.be; +32 3 265 27 06  Public Title: Effect of Pycnogenol® on ADHD.  Scientific Title: Effect of Pycnogenol® on attention-deficit hyperactivity disorder (ADHD): A randomised, double blind, placebo and active product controlled multicentre trial.  Countries of Recruitment: Belgium  Health Condition(s) or Problem(s) Studied: ADHD  Intervention(s): Placebo; Pycnogenol® (standardised, commercially available extract from French maritime pine (*Pinus pinaster*) bark); methylphenidate (slow release standard treatment for ADHD)  Intervention Description: comparison of oral Pycnogenol® (20 or 40 mg/day) to oral methylphenidate hydrochloride (active treatment; 20 or 30 mg/day) and placebo treatment during 10 weeks  Key Inclusion Criteria: 6-12 years; ADHD/ADD  Key Exclusion Criteria: autism, pervasive developmental disorder, schizophrenia, personality disorder, IQ < 70, conduct disorder, dyskinesias, tics or Tourette's syndrome, personal/ family history of psychotic disorder, bipolar illness, depression, or suicide attempt; chronic medical disorder or acute inflammatory disease, glaucoma, heart disease or rhythm disorder, high blood pressure, or peripheral vascular disease; contraindications for the use of methylphenidate (see SmPC) or Pycnogenol® (see Investigator’s Brochure); use of vitamin/ mineral/herbal/omega-3 supplements or other any medication >1 week during the past 3 months. |
|  |  | Study Type: Interventional, randomized, double blind, parallel, phase III trial. Randomisation by software (randomization.com) will be done separately at every trial site (blocks of variable size stratified according to trial site), with a different starting number across trial sites, and taking into account body. For both weight categories, sequentially numbered sealed opaque envelopes will be provided containing the allocation details.  Date of First Enrollment: autumn 2016  Target Sample Size: 144  Recruitment Status: pending  Primary Outcome: Summed ADHD score of the ADHD Rating Scale (ADHD-RS) rated by teachers at the end of intervention (week 10) vs. at the start  Secondary end points:  Summed ADHD score of the ADHD Rating Scale (ADHD-RS) rated by teachers at week 5 compared to the start of intervention  At week 5 and 10 compared to the start of intervention:  Summed ADHD score of the ADHD-RS rated by parents  Summed ADHD score, social behaviour problems and anxiety subscales of the Social-Emotional Questionnaire (SEQ) rated by parents and teachers  Physical and sleep complaints measured by the Physical Complaints Questionnaire (PCQ) rated by parents  At the end (week 10) of intervention (compared to the start):  Percentage of responders, as defined by a reduction of at least 20% of the baseline summed ADHD score of the ADHD-RS  Erythrocyte glutathione (GSH) level (HPLC with coulometric detection)  Urinary 8-hydroxy-2-deoxyguanosine (8-OHdG) level (ELISA)  Plasma malondialdehyde (MDA) level (HPLC with fluorescence detection)  Immune profiling: plasma cytokines and antibody levels, identification of PBMCs and measurement of their functional responses after stimulation (flow cytometry, ELISA), intestinal microbial composition (multiplexed sequencing of 16S ribosomal RNA)  Gene expression quantification: glutathione peroxidase (GPX), catalase (CAT), superoxide dismutase (SOD), xanthine oxidase (XO), Clusterin, Apolipoprotein J (RT-qPCR)  Serum neuropeptide Y (NPY) level (ELISA)  Serum zinc level (AAS)  Lipid soluble antioxidant status (HPLC with coulometric detection)  Urinary catecholamine level (HPLC with coulometric detection)  Antioxidant enzyme level (ELISA) |
| Protocol version | 3 | Pycno2015-14, 2016-09 |
| Funding | 4 | Fund Scientific Research Flanders (FWO), University of Antwerp |
| Roles and responsibilities | 5a | Prof. Dr. Nina Hermans and MSc. Annelies Verlaet  Department of Pharmaceutical Sciences, University of Antwerp  Universiteitsplein 1 (A105, A104), 2610 Wilrijk, Belgium  The University of Antwerp (Laboratory of Nutrition and Functional Food Science) is the sponsor of this trial, with NH being the coordinating investigator. NH and AV are responsible for the analysis of oxidative stress and neurological biomarkers and questionnaire results, as well as data management.  B. Ceulemans, M.D., Ph.D., Neurology, University Hospital Antwerp, Wilrijkstraat 10, 2650 Edegem, Belgium  H. Verhelst, M.D., Ph.D., Paediatric Neurology, University Hospital Ghent  De Pintelaan 185, 9000 Gent, Belgium D. Van West, M.D., Ph.D., University Child & Adolescent Psychiatry, Hospital Network Antwerp, Lindendreef 1, 2020 Antwerp, Belgium |
|  | 5a | As principle investigators, BC, DVW and HV will be primarily responsible for patient inclusion.  H.F.J. Savelkoul, Ph.D., Cell Biology and Immunology Group, Wageningen University, De Elst 1, 6709 PG Wageningen, The Netherlands.  HS is responsible for the analysis of immune biomarkers and genetics. |
|  | 5b | Trial Sponsor: University of Antwerp  Universiteitsplein 1, 2610 Wilrijk, Belgium  Represented by: Prof. Dr. Nina Hermans  Department of Pharmaceutical Sciences, University of Antwerp  Universiteitsplein 1 (A105), 2610 Wilrijk, Belgium +32 3 265 27 32 |
|  | 5c | The University of Antwerp performed the design of the trial, and will be involved in the management, collection, analysis and interpretation of data, as well as manuscript writing and publication (full authority).  Horphag Research, producer of Pycnogenol®, provides material support, without any effect on the preparation or execution of this trial. |
|  | 5d | No Steering Committee, Review Board or Advisory Committee is set up. Decisions regarding evaluation of potential protocol violations concerning the definition of study populations (intention-to-treat; per-protocol, etc.) will be agreed between the CI and the responsible Statistician. Further experts will be involved if necessary. |
| Introduction |  |  |
| Background and rationale | 6a | Methylphenidate (MPH), the standard treatment for ADHD, is prescribed to a large number of children, but causes various side effects. However, ADHD is associated with immune imbalances and increased oxidative stress, which raise potential for nutritional supplements in ADHD therapy.  Pycnogenol®, a polyphenol rich, French maritime pine (*Pinus pinaster*) extract was selected as nutritional supplement in this study, due to its antioxidant, immune modulating and anti-inflammatory effects. In addition, Pycnogenol® was found to have a therapeutic effect in ADHD in a small randomised trial, though this trial had some limitations.  The objective of this study is to evaluate the effect of Pycnogenol® on behaviour and co-morbid physical and psychiatric symptoms, as well as on immunity, neurological parameters, oxidative stress and antioxidant status (to investigate mechanism of action), as compared to placebo and methylphenidate treatment.  The overall frequency rate of adverse side effects due to Pycnogenol® is very low (2.4%), with the majority of adverse effects observed being mild. |
|  |  | The occurrence of adverse effects is unrelated to the dose or duration of use. Gastrointestinal discomfort, dizziness, headache and nausea are the most frequently reported adverse effects. Based on 4 clinical studies on a total of 185 people, Pycnogenol® did not cause any significant changes on systolic or diastolic blood pressure or heart rate. There have been no reports of serious adverse effects in any clinical study or from commercial use since it was initially introduced into the market in Europe around 1970 (ABC Herbalgram, Pycnogenol® Information sheet). |
|  |  | Methylphenidate hydrochloride, the standard treatment for ADHD, has various well known and commonly occurring side effects. |
|  | 6b | Methylphenidate hydrochloride is the standard treatment of ADHD, as active comparator  Placebo, to take into account potential placebo effects |
| Objectives | 7 | Hypotheses:  1. In ADHD therapy, Pycnogenol® is more effective than placebo;  2. In ADHD therapy, Pycnogenol® is not less effective than methylphenidate;  3. As compared to placebo and methylphenidate, Pycnogenol® increases antioxidant levels, reduces oxidative stress, improves immune and neurochemical status and reduces co-morbid physical and psychiatric complaints. |
| Trial design | 8 | Randomized, double blind, parallel, non-inferiority trial |
| Methods: Participants, interventions, and outcomes | | |
| Study setting | 9 | University Hospital Antwerp, Belgium  University Hospital Ghent, Belgium  Hospital Network Antwerp, Belgium |
| Eligibility criteria | 10 | Inclusion: 6-12 years; DSM-IV criteria for ADHD/ADD  Exclusion: Diagnosis of autism, situational hyperactivity, pervasive developmental disorder, schizophrenia, personality disorder, IQ < 70, conduct disorder, dyskinesias, tics or Tourette's syndrome, personal/family history of psychotic disorder, bipolar illness, depression, or suicide attempt; chronic medical disorder or acute inflammatory disease, glaucoma, heart disease or rhythm disorder, high blood pressure, or peripheral vascular disease; contraindications for the use of methylphenidate; use of vitamin/ mineral/herbal/omega-3 supplements or other any medication >1 week during the past 3 months. |
| Interventions | 11a | Patients will receive a dose based on their body weight (1 or 2 oral capsules at breakfast):  • MPH (Medikinet® Retard, methylphenidate hydrochloride modified release): Patients with a body weight < 30 kg will receive 20 mg/day, those with a body weight ≥ 30 kg 30 mg/day. Treatment during the first week always contains 10 mg, increasing 10 mg per week to prevent side effects. |
|  |  | • Pycnogenol®: Patients with a body weight < 30 kg will receive 20 mg/day, those with a body weight ≥ 30 kg 40 mg/day, aiming at a daily dose of 1 mg/kg and taking into account formulation issues [20]. Treatment during the first two weeks always contains 20 mg.  • Placebo: Placebo contains excipients only. |
|  | 11b | In case of patient request, or an adverse event with a possible causal relationship to the use of Pycnogenol® or Medikinet® Retard, trial medication can be discontinued. |
|  | 11c | Adherence, defined as >90% ingestion as scheduled, will be assessed by accountability of investigational products and self-reported adherence. |
|  | 11d | Concomitant medication/supplements during >1 week can lead to drop out. |
| Outcomes | 12 | Primary Outcome: Summed ADHD score of the ADHD Rating Scale (ADHD-RS) rated by teachers at the end of intervention (week 10) vs. at the start  Secondary end points:  Summed ADHD score of the ADHD Rating Scale (ADHD-RS) rated by teachers at week 5 compared to the start of intervention  At week 5 and 10 compared to the start of intervention:  Summed ADHD score of the ADHD-RS rated by parents  Physical and sleep complaints measured by the Physical Complaints Questionnaire (PCQ) rated by parents  At the end (week 10) of intervention (compared to the start):  Percentage of responders, as defined by a reduction of at least 20% of the baseline summed ADHD score of the ADHD-RS  Summed ADHD score, social behaviour problems and anxiety subscales of the Social-Emotional Questionnaire (SEQ) rated by parents and teachers  Erythrocyte glutathione (GSH) level (HPLC with coulometric detection)  Urinary 8-hydroxy-2-deoxyguanosine (8-OHdG) level (ELISA)  Plasma malondialdehyde (MDA) level (HPLC with fluorescence detection) Immune profiling: plasma cytokines and antibody levels, identification of PBMCs and measurement of their functional responses after stimulation (flow cytometry, ELISA), intestinal microbial composition (multiplexed sequencing of 16S ribosomal RNA)  Gene expression quantification: glutathione peroxidase (GPX), catalase (CAT), superoxide dismutase (SOD), xanthine oxidase (XO), Clusterin, Apolipoprotein J (RT-qPCR)  Serum neuropeptide Y (NPY) level (ELISA)  Serum zinc level (AAS)  Lipid soluble antioxidant status (HPLC with coulometric detection)  Urinary catecholamine level (HPLC with coulometric detection)  Antioxidant enzyme level (ELISA) |
|  |  |  |
| Participant timeline | 13 | \|  \| \| **Screening** \| \| **Baseline** \| \|  \| \|  \| \| \| --- \| --- \| --- \| --- \| --- \| --- \| --- \| --- \| --- \| --- \| \| **Evaluations Interventions** \| **Week 0** \| \| **Week 0** \| \| **Week 5** \| \| **Week 10** \| \| \| Inclusion and exclusion criteria \| X \| \| X \| \|  \| \|  \| \| \| Current use of medication/supplements \| X \| \| X \| \| X \| \| X \| \| \| Informed consents \|  \| \| X \| \|  \| \|  \| \| \| Randomization \|  \| \| X \| \|  \| \|  \| \| \| Treatment \|  \| \|  \| \|  \| \|  \| \| \| Treatment distribution \|  \| \| X \| \|  \| \|  \| \| \| Accountability \|  \| \| X \| \|  \| \| X \| \| \| ADHD-RS \|  \| \| X \| \| X \| \| X \| \| \| SEQ \|  \| \| X \| \|  \| \| X \| \| \| PCQ \|  \| \| X \| \| X \| \| X \| \| \| FFQ \|  \| \| X \| \|  \| \| X \| \| \| Blood & urine collection \|  \| \| X \| \|  \| \| X \| \| \| Faeces collection \|  \| \| X \| \|  \| \| X \| \| \| GSH analysis \|  \| \| X \| \|  \| \| X \| \| \| Lipid soluble antioxidants analysis \|  \| \| X \| \|  \| \| X \| \| \| Antioxidant enzyme activity \|  \| \| X \| \|  \| \| X \| \| \| Genetics analysis \|  \| \| X \| \|  \| \| X \| \| \| MDA analysis \|  \| \| X \| \|  \| \| X \| \| \| 8-OHdG analysis \|  \| \| X \| \|  \| \| X \| \| \| Cytokine analysis \|  \| \| X \| \|  \| \| X \| \| \| Antibody analysis \|  \| \| X \| \|  \| \| X \| \| \| PBMC count and reactivity analysis \|  \| \| X \| \|  \| \| X \| \| \| Microbial composition analysis \|  \| \| X \| \|  \| \| X \| \| \| Catecholamine analysis \|  \| \| X \| \|  \| \| X \| \| \| NPY analysis \|  \| \| X \| \|  \| \| X \| \| \| Zinc analysis \|  \| \| X \| \|  \| \| X \| \| \|  \|  \| \|  \| \|  \| \|  \| \| |
| Sample size | 14 | For the estimation of the required sample size, following assumptions were made:  - Patients improve 0,75 SD on the ADHD-RS summed ADHD score as rated by teachers if using Pycnogenol® for 10 weeks, which corresponds to a 20% improvement with active treatment as compared to placebo |
|  |  | - Power of 80%, drop-out of 20%  - Two-sided testing, at a significance level of 0.05 with Bonferroni post-hoc testing correction  Based on these considerations, 48 patients per group will be necessary (n = 144 in total). |
| Recruitment | 15 | The trial population will consist of ADHD and ADD patients of Dr. Ceulemans (UZA), Dr. Van West (ZNA) and Dr. Verhelst (UZ Ghent) and their colleagues, as well as patients from random primary schools in Flanders and “ZitStil” (information centre on ADHD & ADD), which will be invited for this trial by letters. In- and exclusion criteria can be found below. |
| **Methods: Assignment of interventions (for controlled trials)** | | |
| Allocation: |  |  |
| Sequence generation | 16a | Computer-generated random numbers. Randomisation to one of the three treatment groups will be done separately at every trial site (blocks of variable size stratified according to trial site), by means of the website www.randomization.com (original generator, different starting number across trial sites, and taking into account body weights below and above 30 kg). |
| Allocation concealment mechanism | 16b | For both weight categories, sequentially numbered sealed opaque envelopes will be provided containing the allocation details. |
| Implementation | 16c | The allocation sequence will be generated by the participating pharmacies. Physicians will enrol participants, who will receive/choose one of the sealed envelopes in their weight category, containing their randomisation number. With this number, they receive their treatment from the pharmacy. |
| Blinding (masking) | 17a | Only the involved pharmacists know the randomisation codes.  Trial participants, physicians, care providers, outcome assessors and data analysts will be blind. |
|  | 17b | For SUSARs and SAE, the trial treatment is unblinded in the individual trial subject to verify causality before reporting the event to the ethics committee. This means that the trial subject’s treatment is unblinded and the potential SUSARs is reassessed, to decide whether there really is an at least possible causal relationship and whether the event should therefore be classified as a SUSAR. In the event that unblinding occurs, the following should be recorded:  • The randomization number of the unblinded participant  • The reason for unblinding  • The study staff person responsible for unblinding  • A list of person(s) who have been unblinded  If possible, the hospital pharmacy should unblind. In case of emergency/ serious adverse event and in case the pharmacy is not able to unblind, the involved physician can open the sealed envelope. Every involved physician will get the randomisation code in a sealed envelope, only to be used in case of emergency. In case the sealed envelope is opened, this must be notified to the CI and pharmacy immediately, and a new, sealed envelope will be provided. |
|  |  |  |
| **Methods: Data collection, management, and analysis** | | |
| Data collection methods | 18a | Questionnaires will be filled out independently by parents and teachers.  ADHD behaviour will be assessed by parents and teachers by means of the ADHD Rating Scale (ADHD-RS). The ADHD-RS is a validated, internationally accepted rating scale, consisting of the 9 inattention and 9 impulsivity and hyperactivity items based on the DSM-IV. Each item is marked out on a four-point rating scale [[1](#_ENREF_1), [2](#_ENREF_2)]. Parents and teachers will fill out this questionnaire before the start of the intervention, after 5 weeks and at the end of the intervention (10 weeks). A so-called “evaluation of decrement” will take place. Change in ADHD-RS score as rated by teachers and parents will be compared between the three groups. Non-inferiority of Pycnogenol® as compared to MPH will be demonstrated when the difference in effect on ADHD-RS scores (after 10 weeks vs. at baseline) is no more than 5 points [[3](#_ENREF_3)]. This rather wide margin might be justified due to the frequent side effects of MPH (based on literature). Non-inferiority will only be accepted if supported by both intention-to-treat and per protocol analyses [[4](#_ENREF_4), [5](#_ENREF_5)]. Behavioural assessment by teachers is preferred as primary objective due to the higher sensitivity of teachers’ ratings [[6](#_ENREF_6), [7](#_ENREF_7)].  The SEQ, rated twice by parents and teachers, is a behaviour evaluation list to assess core symptoms of social-emotional problems, including frequently occurring psychiatric comorbidities of ADHD. Besides ADHD, three other clusters of social-emotional problems are incorporated in the SEQ (social behaviour problems, anxiety and autism), with items covering the core symptoms of these clusters according to DSM. The SEQ can be used for screening, diagnosis and treatment evaluation. Items are rated on a five-point scale. The reliability and validity of the SEQ are good [[8](#_ENREF_8)].  The Physical Complaints Questionnaire (PCQ), rated twice by parents, has unknown validity and reliability, but includes frequently occurring co-morbid symptoms of ADHD as well as potential side effects. The PCQ was used in research on the effect of nutrition in ADHD before [[9](#_ENREF_9)].  The GSH-method shows good linearity with R = 0.998 and t-test on the intercept showing no statistical difference from zero. The within day RSD (RSD_within_) and the between day RSD (RSD_between_) are 13% and 15%, resp. The overall recovery of standard at concentrations of 0.25; 0.50; 1.00 mM from spiked erythrocytes averaged 98.4% (RSD 8.0%). A recovery t-test at each concentration level does not show any significant difference (p < 0.05) from 100% recovery.  The MDA analysis shows good linearity up to at least 4.80 µM of MDA: R = 0.9998, t-test on the intercept showed no statistical difference from zero. Control plasma samples (0.5 µM MDA-(TBA)2) gave a relative standard deviation (RSD) of 11%, whereas this was less than 5% for higher concentrations of MDA-adduct. For the intermediate precision, five true plasma replicates on 3 different days resulted in a RSD of 10%. The results obtained on 3 different days were not significantly different (ANOVA). A |
|  |  | recovery t-test at each concentration level did not show any significant difference (*P* = 0.05) from 100% recovery.  Analyses of retinol, α -and γ-tocopherol show good linearity up to 4.5, 16.8 and 3.055 µg/ml, RSD_within_ of 11%, 5% and 4%, and RSD_between_ of 10%, 7% and 6%, resp. Recovery was not significantly different from 100% (recovery t-test).  For the other laboratory analyses, linearity, precision and accuracy will be checked before analyses. |
|  | 18b | Two reminders will be sent in case questionnaires are not received within one week after the required date. After every blood and urine collection and in case questionnaires are completed, participants receive two movie tickets. In order to assure analysis of ITT population, it is intended to complete follow-up of all patients, even in case of occurrence of protocol violations which will be documented as well. |
| Data management | 19 | Data management will be performed by UAntwerp. All changes made to the data are documented. The data are backed up daily and only accessible by password. After completion and cleaning of data, the database is locked and exported for statistical analysis. Upon arrival of CRFs at UAntwerp, CRFs are checked for completeness. Data entered into the trial database can be checked by independent staff. Plausibility checks are also conducted. Discrepancies and implausible values are clarified between the data manager and the trial site. The trial site has to answer these queries without unreasonable delay. Full procedures still have to be written. |
| Statistical methods | 20a | Data will be checked for outliers. Missing data will not be accounted for. A drop-out rate of 20% is accounted for.  In the final analysis of the results a so-called “evaluation of decrement” will take place. Change in ADHD-RS score as rated by teachers (primary outcome measure) will be compared between the three groups by means of a one-way ANOVA (categories: group, time; α = 0.05) with post-hoc testing (Tukey). Secondary outcome measures will be analysed following the same procedure. Non-inferiority of Pycnogenol® as compared to Medikinet® Retard will be accepted only if supported by both intention-to-treat and per protocol analyses. |
|  | 20b | Overall analysis as well as separate analyses for subgroups (e.g. based on trial centre, age, gender, presence and severity of ADHD or ADD and (severity of) comorbidities, as well as dietary habits (e.g. whether or not fresh fruit is eaten daily)) will be performed. |
|  | 20c | Missing data will not be accounted for.  Analyses will be conducted on three trial populations:  The primary dataset for analysis is derived from the intention-to-treat (ITT) population. This dataset includes all trial subjects enrolled into the trial and randomized, with the exception of subjects for whom deviations from in- or exclusion criteria were noticed after randomisation. |
|  |  | The secondary dataset for analysis is derived from the per-protocol (PP) population. This dataset includes all trial subjects who were treated according to protocol and adhered to their assigned treatment. Adherence, defined as >90% ingestion as scheduled, will be assessed by accountability of investigational products and self-reported adherence.  The tertiary dataset for analysis is the safety population. This population includes all trial subjects who received any IMP, with analysis as treated. |
| **Methods: Monitoring** | | |
| Data monitoring | 21a | No data monitoring committee (DMC) is set up. The investigators listed above will discuss potential issues at least every six months. |
|  | 21b | No interim analysis is planned.  Premature termination of the trial will be considered if:  • The risk-benefit balance for the trial subject changes markedly  • It is no longer ethical to continue treatment with the IMP  • An unacceptable high number of serious adverse events occurs  • The sponsor/PI considers that the trial must be discontinued for safety reasons  • It is no longer practicable to complete the trial  • A high number of drop-outs (> 40 %)  The sponsor decides on whether to discontinue the trial in consultation with the PI and/or statistician. |
| Harms | 22 | Adverse events will be asked for in the questionnaires filled out at week 5 and 10. Spontaneously reported adverse events will be recorded on the Adverse Event report form. Adverse events will be followed until they resolve. |
| Auditing | 23 | No trial audits are planned yet, but might still be conducted, focussing especially the trial protocol, CRFs, trial subjects’ medical records, drug accountability documentation, and trial-related correspondence. It is unknown yet who will perform this audit. |
| Ethics and dissemination | | |
| Research ethics approval | 24 | Ethical approval has been obtained in UZA (EC 15/35/365), ZNA (EC approval 4656) and UZ Ghent (2016/0969). |
| Protocol amendments | 25 | To ensure that comparable conditions are achieved as far as possible at individual trial sites and in the interests of a consistent and valid data analysis, changes to the provisions of the protocol are not planned. In exceptional cases, however, changes can only be made if agreed by the sponsor, the PI and biometrician, and all Authors of this trial protocol. Any changes to the trial procedures must be made in writing and must be documented with reasons and signed by the Authors of the original trial protocol. Changes will be reported to all investigators, REC/IRBs and trial registries. |
| Consent or assent | 26a | The involved physicians will obtain written informed consent from the participant’s legally accepted representative, after explaining the purpose, risks, potential benefits, etc. |
|  | 26b | Not applicable. |
| Confidentiality | 27 | Personal information about potential and enrolled participants will be collected on sheets by the involved physicians, locked away. Wherever possible, only initials and randomisation numbers are used. Trial subject identification lists at each trial site will be stored separately from trial documentation. All data are only accessible by password/key. |
| Declaration of interests | 28 | None of the investigators has competing interests. |
| Access to data | 29 | The involved statistician(s) as well as researchers/investigators at the University of Antwerp will have access to the final trial dataset, without any reference to the participants’ identity. Contractual agreements are made on the confidentiality and publication of study data. Investigators at each trial site only have access to the data of the participants in that site. |
| Ancillary and post-trial care | 30 | The follow-up observation period is 14 days after the study is completed by the subject. In case of a serious adverse event, the trial code will be broken, the trial medication will be discontinued and the patient will be carefully followed up until the adverse event resolves. Afterwards, though depending on the causality of the serious adverse event, the subject can start standard care for ADHD.  All subjects who have adverse events, whether considered associated with the study intervention or not, must be monitored to determine the outcome. The clinical course of the adverse event will be followed up according to accepted standards of medical practice, even after the end of the period of observation, until a satisfactory explanation is found or the investigator considers it medically justifiable to terminate follow-up. |
| Dissemination policy | 31a | The sponsor plans to publish the trial results in a scientific journal and at Belgian and international congresses. Any published data will observe data protection legislation covering the trial subject and investigators. Success rates or individual findings at individual trial sites are known only to the sponsor.  Also bodies providing any financial or practical support, if any, will be informed about the results of the trial. There will be focus on the broad dissemination of study results. Presentations will be held at info talks of ZitStil in various cities in Flanders as well as in schools. In hospital magazines (e.g. magUZA), results could be described in layman’s terms with emphasis on consequences for ADHD management. For example, if Pycnogenol® supplementation is not inferior to MPH treatment, irrespective of the underlying mechanism(s), leaflets at paediatricians’ practices could inform parents on the advantages of Pycnogenol® in ADHD. If no beneficial behavioural or physical effects would be found, results can still be used to inform on ADHD aetiology. In addition, short research highlights can be published on the university website and social network sites through the UAntwerp Communication Department, as well as in local and national press. |
|  | 31b | Publications will be prepared mainly by the sponsor. The Participating Sites and Investigators agree that they will not:  (A) publish, communicate or otherwise disclose in whatever manner or through any vehicle any information derived from the Study or the Study Data before the clinical trial summary report based on the Study Data and drafted by the Sponsor which (“Study Report”) is notified, if applicable to the competent authorities, to the public, and/or  (B) present abstracts of any information derived from the Study or the Study Data at professional meetings until the Study has been completed and the Study Report has been notified unless a written approval has been obtained from the Sponsor to publish earlier.  The Sponsor will be given the choice to be the first author on any publication. In most instances, the order of the subsequent authors is to be based on recruitment i.e. the number of Study Subjects randomized/enrolled, on Data quality and significant scientific input to the Study or on mutual agreement between the Parties. The Sponsor will retain the right to include in the authorship list, names other than Investigators. |
|  | 31c | There are no plans for granting public access to the full protocol, participant-level dataset, and statistical code. |

| Appendices |  |  |
| --- | --- | --- |
| Informed consent materials | 32 |  |

**Titel: Onderzoek naar Pinus pinaster schorsextract (Pycnogenol®) als alternatieve**

**behandeling voor methylfenidaat bij kinderen met ADHD: een parallele trial.**

FWO MAND 2013 - 11U8314N

Protocolnr.: 2016/09

Organisator: Universiteit van Antwerpen

Onderzoeker: Prof. Dr. Berten Ceulemans

**Inleiding**

Uw kind wordt gevraagd om deel te nemen aan een klinisch-wetenschappelijk onderzoek naar het effect van een plantaardig voedingssupplement (Pycnogenol®, een schorsextract van de Franse pijnboom, Pinus pinaster) op de symptomen van ADHD (attention-deficit/hyperactivity disorder of aandachtstekort–hyperactiviteitstoornis) in vergelijking met de standaardbehandeling voor ADHD (methylfenidaat) en placebo.

Dit onderzoek wordt uitgevoerd omdat resultaten van verschillende studies wijzen op een andere immuunstatus en meer oxidatieve stress bij ADHD patiënten, in vergelijking met gezonde personen. Het immuunsysteem is het verdedigingssysteem van het lichaam met als doel indringers zoals bacteriën en virussen te bestrijden. Oxidatieve stress is een toestand in het lichaam waarbij meer dan een normale hoeveelheid schadelijke stoffen, vrije radicalen genaamd, aanwezig zijn. Deze stoffen beschadigen delen van de cel.

Omdat Pycnogenol® zowel het immuunsysteem kan verbeteren als oxidatieve stress kan verminderen, lijkt dit supplement ideaal voor kinderen met ADHD. Bovendien blijkt uit een voorgaande, kleine studie al dat Pycnogenol ook de symptomen van ADHD kan verbeteren. Verder onderzoek is echter noodzakelijk om een volledig beeld te krijgen van deze effecten, zodat de behandeling van ADHD aangepast en verbeterd kan worden.

De deelname van uw kind aan dit onderzoek is volledig vrijwillig. U kunt besluiten dat uw kind niet zal meedoen en u kunt uw kind later op ieder moment uit het onderzoek terugtrekken.

Voordat u besluit of uw kind deelneemt aan dit onderzoek, moet u weten waarom dit onderzoek wordt uitgevoerd en wat het inhoudt. Lees dit document en stel vervolgens uw vragen aan de onderzoeksarts. Wanneer u de informatie hebt begrepen en u uw kind wilt laten deelnemen aan het onderzoek, moet u dit formulier voor geïnformeerde toestemming ondertekenen voordat uw kind begint met het onderzoek. Als u besluit dat uw kind aan het onderzoek mag deelnemen, krijgt u een exemplaar van dit ondertekende formulier voor uw persoonlijke archief.

Het onderzoek werd goedgekeurd door het onafhankelijke comité voor medische ethiek verbonden aan het Universitaire Ziekenhuis Antwerpen (UZA), na raadpleging van de lokale ethische commissies. Het onderzoek wordt uitgevoerd volgens de richtlijnen voor goede klinische praktijk (ICH/GCP) en de Verklaring van Helsinki (versie 2013) ingesteld ter bescherming van personen die deelnemen aan klinische onderzoeken. In geen geval mag de goedkeuring van het comité voor medische ethiek worden opgevat als aanmoediging om deel te nemen aan dit onderzoek.

**Aard en doel van het onderzoek**

Het doel van het onderzoek is het evalueren van het effect van Pinus pinaster schorsextract (Pycnogenol®) op gedrag, co-morbide symptomen en immuun-, oxidatieve stress- en antioxidant status, in vergelijking met de standaardbehandeling van ADHD (methylfenidaat) en placebo. Wij veronderstellen

• dat behandeling van ADHD met Pycnogenol® beter is dan behandeling met placebo, en niet slechter dan behandeling met methylfenidaat;

• dat Pycnogenol® zorgt voor verbeterde antioxidant waarden, minder oxidatieve stress, verbeterde immuniteit, en minder co-morbide psychiatrische en fysieke symptomen.

Welke behandeling uw kind gedurende dit onderzoek (10 weken) krijgt, Pycnogenol®, methylfenidaat of placebo, kunt u niet kiezen. Aan het begin en het einde van deze 10 weken zullen een bloed-, feces- en urinestaal worden afgenomen. Op dezelfde tijdspunten en na 5 weken moeten ook een aantal vragenlijsten worden ingevuld. Gemiddeld hebben ouders 30 min. nodig voor het invullen van de vragenlijsten. Na 5 weken zult u ook kort telefonisch worden gecontacteerd. Indien na deze 10 weken blijkt dat uw kind in de methylfenidaat- of placebogroep terechtkwam, kan u wel gratis Pycnogenol® supplementen krijgen voor 10 weken behandeling (op eigen initiatief en verantwoordelijkheid). Dit onderzoek is bedoeld voor kinderen tussen 6 en 12 jaar oud met een diagnose van ADHD of ADD. Deelnemers mogen geen chronische aandoeningen (bv. diabetes, auto-immuunziekte) hebben. Bovendien mag er geen gebruik gemaakt zijn van ADHD medicatie of voedingssupplementen in de voorbije 3 maanden gedurende langer dan één week. De in dit onderzoek verzamelde informatie zal helpen bij het bepalen van een nieuwe, op voedingssupplementen gebaseerde behandeling van ADHD.

Kinderen die in aanmerking komen voor dit onderzoek en ervoor kiezen deel te nemen zullen tweemaal een bloedafname ondergaan en tweemaal gevraagd worden een feces- en urinestaal te voorzien. Deze bloedafnames omvatten ook een afname van DNA zodat effecten van genen onderzocht kunnen worden.

**Procedures**

Er zullen ongeveer 144 kinderen met ADHD of ADD aan het onderzoek deelnemen, verdeeld over verschillende centra (Universitair Ziekenhuis Antwerpen, België, Ziekenhuis Netwerk Antwerpen, België, Revalidiatiecentrum Noorderkempen in Wuustwezel, en verschillende zelfstandige psychiaters), met de mogelijkheid tot uitbreiding naar andere landen en locaties indien nodig. Deelname aan dit onderzoek vereist het strikt volgen van de opgelegde behandeling (Pycnogenol®, methylfenidaat of placebo) gedurende 10 weken, twee bloed-, feces- en urinestalen en het driemaal invullen van een aantal vragenlijsten. De eerste staalafname kan, maar moet niet, gebeuren tijdens een reguliere consultatie, samen met het invullen van de eerste vragenlijsten. Een bezoek aan het onderzoekscentrum 10 weken na de start van het onderzoek is vereist. Ook de leerkracht van uw kind wordt gevraagd vragenlijsten in te vullen.

Als u en uw kind akkoord gaan met deelname en dit formulier voor geïnformeerde toestemming ondertekenen, zullen de volgende procedures worden uitgevoerd, o.a. om vast te stellen of uw kind in aanmerking komt voor deelname aan het onderzoek.

- Beoordeling van de geschiktheidscriteria voor het onderzoek

- Beoordeling van de huidige medicatie

- Beoordeling algemene gezondheid

- Beoordeling van de medische voorgeschiedenis aan de hand van het medisch dossier;

Als de onderzoeksarts na deze procedures van mening is dat uw kind aan het onderzoek kan deelnemen, worden de volgende procedures uitgevoerd:

- Een lichamelijk onderzoek (lengte en gewicht);

- Het invullen van vragenlijsten rond ADHD symptomen, fysieke en mentale symptomen (bv. hoofdpijn, buikpijn en angstig zijn), voedingsgewoonten, allergie en infecties, en problemen tijdens de zwangerschap;

- Bloed-, feces- en urinestalen, inclusief afname van DNA. De hoeveelheid bloed die wordt afgenomen, bedraagt 16 ml.

Tien weken na de start van het onderzoek worden deze procedures opnieuw uitgevoerd. Vijf weken na de start van het onderzoek worden extra vragenlijsten ingevuld (korte versie, 20min.) en zult u kort telefonisch worden gecontacteerd.

**Risico’s en ongemakken**

Bij het afnemen het bloedstaal kan uw kind enig ongemak ervaren en kan er een kleine bloeding, verkleuring of een blauwe plek ontstaan waar de naald is ingebracht. Er kunnen bloedpropjes of infecties ontstaan op de prikplaats, maar dit is zeer zeldzaam. Uw kind kan flauwvallen tijdens of net na het afnemen van bloed. Als uw kind zich slap voelt, moet het direct gaan liggen om te voorkomen dat het zich bezeert bij een val. Het onderzoekspersoneel moet worden gewaarschuwd. De totale hoeveelheid bloed die van uw kind wordt afgenomen tijdens het onderzoek bedraagt telkens minder dan 1 eetlepel.

Pycnogenol geeft zelden bijwerkingen, maar kan duizeligheid, hoofdpijn, geïrriteerdheid, intestinale symptomen en aften in de mond veroorzaken, en kan symptomen van auto-immuunziekte versterken. Een supplementatiestudie bij kinderen met ADHD vermeldt enkel bijwerkingen bij twee van de 44 deelnemers (traagheid en matige last aan de maag) (Trebaticka et al, 2006).

Methylfenidaat, de standaardbehandeling bij ADHD, heeft een aantal frequent voorkomende bijwerkingen, zoals hoofdpijn, buikpijn, verminderde eetlust en slapeloosheid.

**Voordelen**

De voordelen van deelname aan dit klinisch-wetenschappelijke onderzoek zijn verschillende medische onderzoeken en nauwkeurige observatie van de gezondheid van uw kind door een onderzoeksarts. De gezondheid van uw kind heeft mogelijk niet direct baat bij deelname aan dit onderzoek. Toekomstige proefpersonen kunnen baat hebben bij de uit dit onderzoek verkregen informatie.

**Betaling voor deelname**

U wordt niet betaald voor deelname van uw kind aan dit klinisch-wetenschappelijk onderzoek. Uw kind krijgt na de bloedafname wel twee filmtickets mee naar huis. De instelling van uw arts wordt door het onderzoeksfonds vergoed voor de specifieke verrichtingen en uitgaven in verband met het uitvoeren van dit onderzoek.

**Vertrouwelijkheid**

Uw kind heeft het recht op vertrouwelijkheid en alle informatie die verzameld wordt voor deze studie is vertrouwelijk conform de wet ter bescherming van de persoonlijke levenssfeer en de rechten van de patiënt. Zijn/haar naam, adres, telefoonnummer, of andere persoonsgegevens (bv. sociaal zekerheidsnummer) zullen niet worden bekendgemaakt.

Zijn/haar gegevens, zoals resultaten van de studie en informatie over zijn/haar algemene gezondheidstoestand, zullen door de arts worden verzameld en worden overgemaakt aan de Universiteit van Antwerpen (UAntwerpen), waar ook de resultaten van de tests die werden uitgevoerd, bewaard worden.

Uw kind zal alleen worden geïdentificeerd met een uniek codenummer en de informatie over de code zal worden bewaard op een veilige plaats die alleen toegankelijk is voor het studiepersoneel. Alleen de studiearts en het studiepersoneel zullen toegang hebben tot deze codelijst en deze zo nodig kunnen decoderen als bijkomende informatie noodzakelijk is.

U hebt het recht de studiearts te vragen welke gegevens over uw kind werden verzameld en wat de functie ervan is in het kader van de studie. U hebt het recht op toegang en correctie van de persoonsgegevens (demografische gegevens) in het geval ze niet correct zijn. Volgens de wet van 8 december 1992 (gewijzigd door de wet van 11 december 1998) gevolgd door de richtlijn 95/46/EC van 24 oktober 2002 ter bescherming van de persoonlijke levenssfeer en de patiëntenrechten (conform de wet van 22 augustus 2002) die voorziet in een ombudsdienst waar de patiënt een klacht kan indienen betreffende de uitoefening van zijn rechten, hebt u recht tot inzage en correctie van uw kind zijn/haar persoonsgegevens.

De medische gegevens van uw kind zullen niet door UAntwerpen worden bekend gemaakt. Om het even wanneer tijdens of na de studie echter, zal het personeel van UAntwerpen of de organisatie verantwoordelijk voor de opvolging af en toe toegang worden verleend tot zijn/haar medische gegevens i.v.m. de studie om de correctheid van de tijdens de studie verzamelde informatie te bevestigen. Het onderzoek van het medisch dossier van uw kind door derden (bv. UAntwerpen, ethische commissie, inspecteur van regelgevende instanties) gebeurt uitsluitend onder de verantwoordelijkheid van de studiearts of een van zijn medewerkers. De personen die toegang hebben tot het dossier zijn gehouden aan het beroepsgeheim.

Computerbestanden die verzamelde gegevens hebben, zullen tegen misbruik worden beveiligd.

De verzamelde gegevens zullen worden geanalyseerd door UAntwerpen om de effecten van Pycnogenol® op gedrag, co-morbide symptomen en immuun- en oxidatieve stress status te vergelijken met die van methylfenidaat en placebo. De gegevens van uw kind en die van de andere patiënten die aan de studie deelnamen, kunnen worden gebruikt voor bepaalde statistische analyses. UAntwerpen kan de resultaten van deze studie dan overmaken aan gezondheidsinstanties over de hele wereld en de resultaten kunnen ook worden gebruikt in studierapporten of voor wetenschappelijke presentaties op wetenschappelijk of medische bijeenkomsten of worden gepubliceerd in wetenschappelijke tijdschriften. De naam van uw kind zal niet bekend gemaakt worden in verslagen over dit onderzoek. De resultaten kunnen worden gebruikt voor later medisch onderzoek.

Daarenboven is het niet ondenkbaar dat UAntwerpen later bijkomende gegevens uit zijn/haar medisch dossier/medische gegevens opvraagt om al verzamelde gegevens in de juiste medische context te plaatsen.

In overeenstemming met de nationale voorschriften hebt u het recht het verzamelen van bijkomende analyses te weigeren.

Indien u beslist uw toestemming tot deelname van uw kind te herroepen zullen geen nieuwe gegevens meer aan de database worden toegevoegd. Echter, in overeenstemming met de nationale voorschriften kan tot dan verzamelde informatie nog aan UAntwerpen worden verstrekt.

De studiearts zal uw huisarts op de hoogte brengen van de deelname van uw kind aan de studie, als u dat wenst.

**Vergoeding voor letsel**

Het risico voortvloeiend uit deelname aan dit onderzoek is gedekt conform artikel 29 van de wet van 7 mei 2004 betreffende experimenten op menselijke proefpersonen. Volgens dit artikel is UAntwerpen, zelfs indien foutloos, aansprakelijk voor de schade die berokkend werd aan de deelnemer of zijn wettelijke vertegenwoordigers en die rechtstreeks of onrechtstreeks verband houdt met de studie. UAntwerpen heeft een verzekering afgesloten die deze aansprakelijkheid dekt (Algemeen UAntwerpen verzekeringcontract voor wetenschappelijke studies).

Het ondertekenen van dit formulier houdt niet in dat u afstand doet van uw wettelijke rechten waar u anders als deelnemer aan een onderzoeksstudie recht op hebt. Voor bijkomende informatie over de verzekeringsdekking kunt u bij uw arts terecht.

**Kosten**

Tijdens deelname van uw kind aan dit onderzoek zijn de behandelingen, lichamelijke onderzoeken, bloed- en urinetesten, en verwerking van de vragenlijsten en medische voorgeschiedenis niet ten laste van de deelnemer.

**Vrijwillige deelname**

Uw beslissing om uw kind al dan niet aan dit onderzoek te laten deelnemen, is volledig vrijwillig. U kunt besluiten uw kind niet te laten deelnemen en u kunt uw kind op ieder moment uit het onderzoek terugtrekken. Een dergelijke beslissing zal niet leiden tot verlies van voordelen waar uw kind anders recht op heeft. Als u besluit uw kind uit het onderzoek terug te trekken, wordt u verzocht om de onderzoeksarts hiervan op de hoogte te stellen.

**Meer informatie**

Als u vragen hebt over dit onderzoek kunt u deze stellen aan de coördinator van het onderzoek (03 265 27 06). Als u later nog vragen hebt, kunt u bellen met Prof. Dr. Berten Ceulemans op 03 821 57 60.

Als u vragen hebt over de rechten van de deelnemers aan een klinisch-wetenschappelijk onderzoek, kunt u bellen met de ombudsdienst van Universitair Ziekenhuis Antwerpen op 03 821 31 60.

Indien u van mening bent dat uw kind letsel heeft opgelopen als gevolg van de deelname aan dit onderzoek, kunt u bellen met de ombudsdienst van Universitair Ziekenhuis Antwerpen op 03 821 31 60.

Voor noodgevallen is er iemand beschikbaar op 02 831 30 00 (24 uur per dag). U kunt ook zelf naar de dienst Spoedgevallen gaan.

**Toestemming voor deelname aan het ADHD onderzoek.**

**Titel: Onderzoek naar Pinus pinaster schorsextract (Pycnogenol®) als alternatieve**

**behandeling voor methylfenidaat bij kinderen met ADHD: een parallele trial.**

FWO MAND 2013 - 11U8314N

Ondergetekende ouder/voogd, verklaart hierbij de goedkeuring te verlenen in naam van beide ouders/voogden.

Proefpersoon ____________________ Geboorte- ____________

(familienaam, voornaam) datum (DD/MM/JJJJ)

Onderzoeker/ ____________________ Proefpersoon- ____________

vertegenwoordiger (familienaam, voornaam) nummer

Ik heb de informatie in het formulier voor geïnformeerde toestemming voor de proefpersonen gelezen en alle relevante aspecten van het onderzoek besproken met de onderzoeksarts of onderzoeks-medewerkers, waaronder vergoeding in geval van aan het onderzoek gerelateerd letsel en de goedkeuring door de institutionele beoordelingscommissie. Al mijn vragen over het onderzoek werden naar mijn tevredenheid beantwoord. Ik begrijp dat ik mijn kind op ieder moment uit het onderzoek kan terugtrekken of kan weigeren aan procedures deel te nemen zonder dat de doorlopende klinische zorg voor mijn kind daardoor wordt beïnvloed. Ik stem er door ondertekening van dit formulier in toe, dat mijn kind als proefpersoon deelneemt aan dit onderzoek.

Door ondertekening van dit formulier voor geïnformeerde toestemming geef ik toestemming aan vertegenwoordigers van UAntwerpen, bevoegd personeel van het Universitair Ziekenhuis Antwerpen, en de bevoegde autoriteiten of ethische commissie tot rechtstreekse inzage van het originele medische dossier van mijn kind ter controle van de procedures in het kader van het klinisch-wetenschappelijk onderzoek en/of gegevens, zonder de vertrouwelijkheid van mijn kind te schenden, voor zover dat voor de relevante wet- en regelgeving wordt toegestaan.

Door ondertekening van dit formulier voor geïnformeerde toestemming heb ik geen afstand gedaan van enig wettelijk recht dat mijn kind anders zou hebben als deelnemer aan een klinisch-wetenschappelijk onderzoek.

Gelieve de onderzoeksarts of een van de medewerkers een seintje te geven wanneer

deelname aan de studie of inname van de studiebehandeling gestopt wordt.

______________________ ______________________ ____________

Handtekening ouder/voogd Naam in blokletters Datum

Ο Ik wil niet dat de bloed-, urine-, en stoelgangstalen van mijn kind later gebruikt

kunnen worden voor eventueel bijkomende onderzoeken.

Ik begrijp dat de bloedafname voor dit klinisch-wetenschappelijk onderzoek ook een afname van DNA omvat en geef hiervoor toestemming.

______________________ ______________________ ____________

Handtekening ouder/voogd Naam in blokletters Datum

Ik verklaar dat de onderzoeksstudie door mij of mijn onderzoekspersoneel aan bovenvermelde patiënt werd uitgelegd, inclusief het doel, de procedures en de aan deze onderzoeksstudie verbonden mogelijke risico’s en potentiële voordelen. Alle vragen werden tot voldoening van de persoon beantwoord. Ik verklaar te werken in overeenstemming met de ethische principes van de verklaring van Helsinki en de Belgische wet van 7 mei 2004 betreffende experimenten op menselijke proefpersonen.

De persoon die dit formulier voor geïnformeerde toestemming ondertekent, krijgt een ondertekend exemplaar.

______________________ ______________________ ____________

Handtekening onderzoeker/ Naam in blokletters Datum

vertegenwoordiger

|  | | |
| --- | --- | --- |
| Biological specimens | 33 | In order to analyse the proposed biomarkers (GSH, MDA, lipid soluble antioxidants, 8-OHdG, immune markers like antibodies/cytokines/cells, genetics, …: see 13), 16 ml venous blood (in K3 EDTA tubes and 1 serum tube) will be collected at the start and the end of intervention, as well as urine samples. Faeces samples will be collected from subgroups (n = 60 in total). Samples will be coded with the participants’ randomisation number. |

*It is strongly recommended that this checklist be read in conjunction with the SPIRIT 2013 Explanation & Elaboration for important clarification on the items. Amendments to the protocol should be tracked and dated. The SPIRIT checklist is copyrighted by the SPIRIT Group under the Creative Commons “[Attribution-NonCommercial-NoDerivs 3.0 Unported](http://www.creativecommons.org/licenses/by-nc-nd/3.0/)” license.

**References**

1. D’Andrea G. Pycnogenol: A blend of procyanidins with multifaceted therapeutic applications? Fitoterapia. 2010;81:724-36.

2. Döpfner M, Steinhausen H-C, Coghill D, Dalsgaard S, Poole L, S.J. R et al. Cross-cultural reliability and validity of ADHD assessed by the ADHD Rating Scale in a pan-European study. Eur Child Adolesc Psychiatry. 2006;15:I/46–I/55.

3. Berek M, Kordon A, Hargarter L, Mattejat F, Slawik L, Rettig K et al. Improved functionality, health related quality of life and decreased burden of disease in patients with ADHD treated with OROS® MPH: is treatment response different between children and adolescents? Child Adolesc Psych Mental Health. 2011;5(26).

4. Christensen E. Methodology of superiority vs. equivalence trials and non-inferiority trials. J Hepatol. 2007;46:947-54.

5. Piaggio G, Elbourne DR, Altman DG, Pocock SJ, Evans SJ. Reporting of noninferiority and equivalnce randomized trials: an extension of the CONSORT statement. JAMA. 2006;295:1152-60.

6. Power TJ, Doherty BJ, Panichelli-Mindel SM, Karustis JL, Eiraldi RB, Anastopoulos AD et al. The Predictive Validity of Parent and Teacher Reports of ADHD Symptoms. J Psychopathol Behav Assessment. 1998;20(1):57-81.

7. Tripp G, Schaughency EA, Clarke B. Parent and Teacher Rating Scales in the Evaluation of Attention-deficit Hyperactivity Disorder: Contribution to Diagnosis and Differential Diagnosis in Clinically Referred Children. J Developm Behav Ped. 2006;27(3):209-18.

8. TestWeb. Sociaal-Emotionele Vragenlijst (SEV). Bohn Stafleu van Loghum, Springer Media. 2013 <http://testweb.bsl.nl/tests/sev/>.

9. Pelsser LMJ, Frankena K, Toorman J, Savelkoul HFJ, Dubois AE, Pereira RR et al. Effects of a restricted elimination diet on the behaviour of children with attention-deficit hyperactivity disorder (INCA study): a randomised controlled trial. Lancet. 2011;377:494-503.
